# Comparison of the Relationship Between SI and RASI Scores With the Outcome of Sepsis Patients

**DOI:** 10.3389/fmed.2022.872725

**Published:** 2022-06-29

**Authors:** Amir Masoud Hashemian, Zahra Baghshani, Roohie Farzaneh, Hamid Zamani Moghadam, Fatemeh Maleki, Farhad Bagherian, Somayyeh Ahmadnezhad, Mahdi Foroughian

**Affiliations:** ^1^Department of Emergency Medicine, Faculty of Medicine, Mashhad University of Medical Sciences, Mashhad, Iran; ^2^Department of Emergency Medicine, Mashhad University of Medical Sciences, Mashhad, Iran; ^3^Department of Emergency Medicine, Faculty of Medicine, Birjand University of Medical Sciences, Birjand, Iran; ^4^Department of Emergency Medicine, Faculty of Medicine, Babol University of Medical Sciences, Babol, Iran; ^5^Department of Emergency Medicine, Faculty of Medicine, Mazandaran University of Medical Sciences, Sari, Iran

**Keywords:** SI, RASI, sepsis, emergency, infection

## Abstract

The aim of this study was to compare the relationship between shock index (SI) and respiratory adjusted shock index (RASI) scores with the final outcome of sepsis patients referred to the emergency department. This was prospective research that examined individuals who had been diagnosed with sepsis, determined by the presence of at least two of the three quick sepsis-related organ failure assessment (qSOFA) criteria and the presence of an infectious disease based on a diagnosis made by a hospital physician of Imam Reza and Ghaemshahr of Mashhad in 2019. Demographic information of patients, SI score, RASI score, and information related to the patient's clinical symptoms were recorded in the checklist. The final outcome of this study was considered mortality. Data analysis was performed using descriptive and inferential tests. In the present study, a total of 178 patients, 46 patients (25.8%) were transferred to the intensive care unit, and 98 patients (55.1%) were admitted to the normal wards. Eighty-five patients (47.75%) died and the mean length of hospital stay of all patients was 11.07 ± 9.23 days. Forty-four patients (24.7%) had referred with a decreased level of consciousness and 44 patients (24.7%) presented with confusion. The rest of the patients reported normal levels of consciousness. Kaplan Mir analysis with log-rank was performed to determine the difference in survival distribution in different SI groups: Survival distribution was not statistically different for the four defined groups (based on statistical quartiles (*P* = 0.320). Receiver operator curves were considered as the date of death in the case of the deceased and the date of discharge from the hospital in the case of the living as censored. The AUC of the RASI scoring system for predicting mortality was 0.614 (*P* = 0.009) while this value was not significant for SI (*P* = 0.152). In logistic regression analysis, it was found that by adjusting for the variables of age, sex, sepsis etiology, blood pressure and heart rate, level of consciousness, and gender, patients with the lower respiratory rate (OR 1.6, z = −0.159 *p* = 0.007), younger age (OR 1.6, z = −0.029 *p* = 0.006) and higher RASI score are more in risk of mortality (OR 1.29, z = 1.209, *p* = 0.031). The results of our study showed that RASI scoring can be a good criterion for predicting the chance of mortality in patients with sepsis and could be used complementary to previous criteria such as SI. Patients with high RASI scores should be given more attention to reducing the chance of death.

## Introduction

Sepsis is a systemic reaction of the body to invasive microorganisms such as bacteria and fungi and is one of the diseases that patients admitted to different parts of the hospital may be infected with (1). Sepsis is the second greatest cause of mortality among admitted patients with a variety of illnesses, and one of the top 10 causes of mortality in all inpatients (2). Sepsis is more common in the elderly and significantly affects people with cancer and defective immune systems. So that in its most acute form, the infection disrupts several organs of the body and creates critical conditions (4). Rhabdomyolysis has been reported in bacterial, viral, and fungal infections (5). Sepsis-induced hypoxia, bacterial invasion of myocytes, decreased activity of glycolytic, oxidative, lysosomal enzymes, and endotoxin-induced damage all lead to rhabdomyolysis during infections. Legionella is the most common cause of rhabdomyolysis due to sepsis (6). The host's reaction to infection is sepsis. The invading agent and the host body's activated inflammatory mediators impair the body's defensive and regulatory systems, causing the body's homeostasis to be disrupted. The most frequent primary signs of the systemic response, also known as the systemic inflammatory response syndrome, are tachycardia, tachypnea, fever or hypothermia, and immune system activation (leukocytosis or leukopenia) (SIRS) (7). The definition of SIRS based on quick SOFA criteria is that the patient has at least two of the following three criteria: (1). The respiratory rate of higher than 22 times per minute or more, (2). The change in consciousness and (3). Systolic blood pressure 100 mmHg or less. When SIRS is verified or presumed to be caused by bacteria, it is referred to as sepsis. Similarly, if sepsis is affiliated with one or more organ dysfunction signs, such as hypoperfusion, hypotension, metabolic acidosis, acute mental state change, oliguria, or ARDS, it is referred to as severe sepsis and is referred to as septic shock with hypotension which does not adapt to intravenous fluids and interrupts organ dysfunction or contributes to perfusion impairment (7). The consequences of sepsis have greatly improved, probably because of the focus on early diagnosis and the rapid and timely administration of effective antibiotics, and advances such that early detection of the disease is a major challenge (3). In the early stages, the diagnosis of sepsis from non-infectious conditions, especially in critically ill patients is difficult and diagnosis, treatment, and its results are significantly different among patients with sepsis and without sepsis (6). Notwithstanding the advent of new explanations on the origin and pathogenesis of sepsis, as well as the development of extremely powerful antibiotics and antifungal agents, there has been little progress in decisively lowering mortality from this syndrome (3). One of the most essential issues in this respect is the establishment of precise procedures for diagnosing the outcomes in patients with sepsis, particularly critically ill individuals. A basic scoring system for measuring shock and hemodynamics in patients is the SI score (8). Recent studies have shown the importance of tachycardia in predicting cardiac arrest and as an indicator of organ dysfunction; For this reason, a new criterion was defined with the aim of including the RR effect in shock prediction called RASI, which is calculated according to the formula RR / 10 × HR / SBP (9, 10). Jiang et al. (10) evaluated 360 individuals with sepsis in research aiming at employing RASI to detect latent shock and quality of care in sepsis patients. Lactate (OR 1.55, z = 4.38, p0.0001) and RASI (OR 2.27, z = 3.03, p0.002) were shown to indicate the need for more care in regression analysis. For shock detection, the AUCs for RASI, SI, and qSOFA were 0.71, 0.6, and 0.61, respectively. In contrast to SI (0.64) and qSOFA, RASI exhibited a substantial AUC of 0.75 in identifying the degree of care (0.62). They concluded that RASI might be effective as a quick-response method for forecasting critical diseases in sepsis patients (10). In a retrospective study, Caputo et al. (9) examined the RASI criteria for determining the presence of latent shock in trauma patients. A total of 3,093 patients participated in this study. In terms of the SI index, there was no significant difference between discharged and hospitalized patients' rates [0.6 (95% CI, 0.5–0.7) vs. 0.7 (95% CI, 0.5–0.8)]. However, in the study of the RASI index, a significant difference was observed between discharged and hospitalized patients [1.1 (95% CI, 1.04–1.18) vs. 1.46 (95% CI, 1.35–1.55)]. The range under the ROC curve was 0.58 for the SI score and 0.94 for the RASI score. They concluded that the RASI score improves diagnostic accuracy for detecting latent primary shock in trauma patients compared with SI (9). One of the most essential issues in this respect is the establishment of precise techniques for assessing the prognosis of patients with sepsis, particularly critically sick patients, as well as the kind of therapy and prioritization of patient care. As a result, we decided to look into the link between RASI (Respiratory adjusted shock index) and the final outcome of sepsis patients who were brought to the emergency room.

## Methods

This was prospective research conducted on individuals having an initial impression of sepsis (depending on the existence of at least two of the three qSOFA signs and the existence of an infectious condition based on a hospital physician's diagnosis) who were seen in Mashhad city's emergency departments. The purpose of the study was explained to the patients and their consent or that of their companions was taken to participate in the study. Demographic information of patients including gender, age, medical history, and information related to the patient's clinical signs were collected in a checklist by the resident.

The present study was conducted during three main phases, each of which is referred to below:

1- Data collection and preprocessing according to the parameters required to calculate SI and RASI in patients with sepsis, including heart rate, systolic blood pressure, and respiratory rate. After preparing a comprehensive form regarding the desired parameters, the residents of emergency medicine were asked to complete and submit the relevant information.

2- Performing calculations related to determining the score of SI (HR / SBP) and RASI (RR / 10 × HR / SBP) for each patient according to the mentioned formulas.

3- Evaluating and analyzing the obtained data and comparing SI and RASI scores in determining the final outcome of patients, including 1- Mortality during hospitalization 2- Type of admission of patients, in the emergency department, ward, ICU, or discharge; 3- the duration of hospitalization.

Data analysis was performed using descriptive statistics in SPSS software version 20. The characteristics of the subjects were presented by descriptive statistical methods including central indicators, dispersion, and frequency distribution in the form of appropriate tables and graphs. *T*-test was used to compare quantitative variables in case of normal distribution of data and the Mann-Whitney test was used otherwise. Survival analysis was used to investigate the relationship between the scales and the incidence of mortality. ROC analysis to check the AUC of each score was performed. We used STATA version 17 to perform Delong's test to compare AUC of scores. Logistic regression model was used to adjust for other contextual and clinical variables. Kendall tau Rank Correlation (v1.0.13) was used to show the correlation diagram. In all calculations, a value of 0.05 was considered significant.

## Result

In the present study, a total of 178 patients with sepsis were studied. Of these, 101 (56.7%) were male and 77 (43.3%) were female. The mean age of these individuals was 68.41 ± 17.35 years. Regarding the source of infection, the final diagnosis was pneumo-sepsis in 154 patients (86.51%). Eighteen patients (10.11%) had urosepsis. Cellulite and catheter infections were seen in two patients (1.12%). Diarrhea was read as a cause of sepsis in one patient (0.6%). One patient had both pneumo-sepsis and urosepsis (Table 1).

**Table 1:** Demographic data and clinical parameters.

		***n***	**%**
**Sex**	Male	101	56.74
	Female	77	43.26
**Source of sepsis**	Pneumo-sepsis	154	86.52
	Urosepsis	18	10.11
	Cholangin	2	1.12
	Cellulitis	2	1.12
	Catheter related infection	2	1.12
	Gastroenteritis	1	0.56
**Level of consciousness**	Normal	90	50.56
	Decreased	44	24.72
	Confusion	44	24.72
**Admission type**	ICU	46	25.84
	Ward	98	55.06
	ED	34	19.1

Forty-four patients (24.7%) were referred with a decreased level of consciousness and 44 patients (24.7%) presented with confusion. The rest of the patients reported normal levels of consciousness.

Finally, 46 patients (25.8%) were transferred to the intensive care unit. Ninety-eight patients (55.1%) were admitted to normal wards. Thirty-four people (19.1%) were treated in the emergency department. Hemodynamic status of patients are shown in Table 2.

**Table 2:** Hemodynamic status of patients.

	**Mean**	**SD**	**Median**	**Q1**	**Q3**
**RR**	30.32	8.59	29.5	24	36
**PR**	112.78	21.98	110	103	124.25
**SBP**	122	29.3	122.5	100	144.25

**Figure 1:**
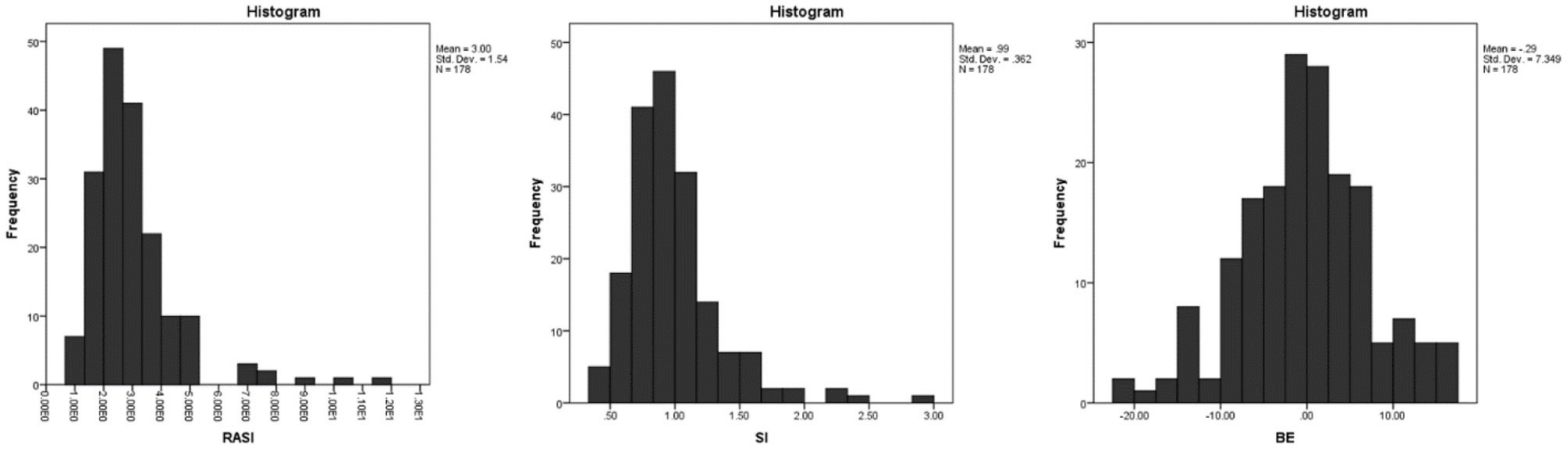
Frequency of different values of BE, RASI and SI.

**Figure 2:**
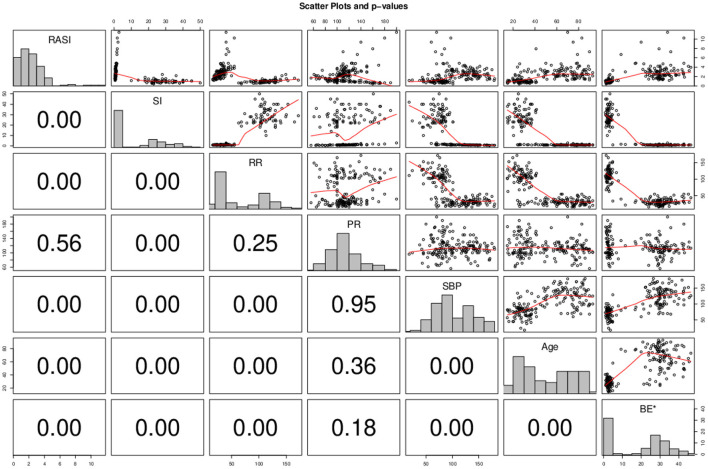
Correlation matrix of study variables. *P-*value is shown numerically on the left side of the matrix. * To display BE numerical values in the graph due to the negative number of data, all values are added with a fixed value. X border and corresponding border in front is belonging to the first variable in each histogram and the Y and the border in front is belonging to the second variable of the correlation test.

RASI, SI, and BE estimates are calculated and presented in Figure 1. The relationship between the main variables of the study with hemodynamic status, age and with each other was measured using the Spearman correlation test and shown in Figure 2.

RSAI correlated significant inverse correlation with SI (Spearman rho = −0.555; *P* < 0.001), significant inverse correlation with RR (Spearman rho = −0.486; *P* < 0.001), Significant direct correlation with SBP (Spearman rho = + 0.467; *P* < 0.001), significant direct correlation with age (Spearman rho = + 0.58; *P* < 0.001) and a direct significance correlation with BE (Spearman rho = + 0.711; *P* < 0.001). SI had a direct correlation with RR (Spearman rho = + 0.719; *P* < 0.001), significant direct correlation with PR (Spearman rho = + 0.240; *P* = 0.00012), Significant inverse correlation with SBP (Spearman rho = −0.841; *P* < 0.001), significant inverse correlation with age (Spearman rho = −0.719; *P* < 0.001) and There was a significant inverse correlation with BE (Spearman rho = −0.750; *P* < 0.001). BE had a significant inverse correlation with RR (Spearman rho = −0.682; *P* < 0.001), weak significant direct correlation with SBP (Spearman rho = + 0.005; *P* < 0.001) and had a significant inverse correlation with age (Spearman rho = −0.631; *P* < 0.001).

Then, the relationship between the study variables and the final outcome of mortality and length of hospital stay was investigated. Of the total population, 85 (47.75%) died. The mean duration of hospitalization was 11.07 ± 9.23 days. The last day of hospitalization was considered the date of death in the case of the deceased and the date of discharge from the hospital in the case of the living was censored.

**Figure 3:**
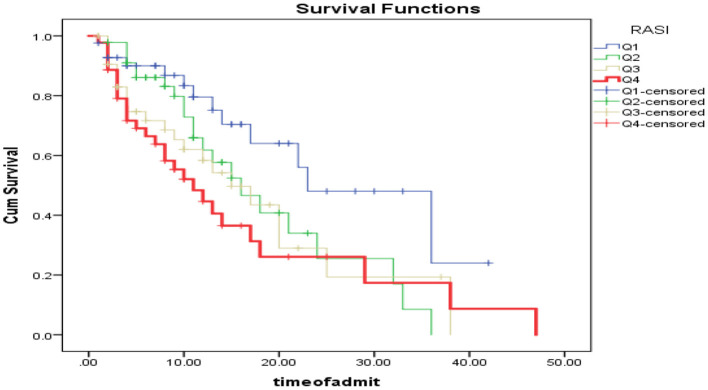
Patient survival analysis based on RASI quarters.

As shown in Figure 3, patients were categorized based on RSAI quartiles into 4 categories of <2.07 (Q1), between 2.07 and 2.71 (Q2), between 2.71 and 3.48 (Q3) and more than 3.48 (Q4). Kaplan Mir analysis with log-rank was performed to determine the difference in survival distribution in different RSAI groups: The survival distribution was statistically significantly different for the four defined groups, χ2 (3) = 9.76, *P* < 0.0005. As shown in Table 3, One-way ANOVA of mean survival of patients was compared based on the quartiles of RASI, SE, and BE. It was found that the mean survival of patients having RASI within Q1 ranges was significantly higher than those within Q4 range (*P* = 0.014); while other groups had no significant difference in case of RASI (*P* > 0.05). Also, these comparisons were not statistically significant for SE and BE (*P* > 0.05).

**Table 3:** Mean survival of live and deceased patients.

		***n* Total**	***n* Death (%)**	**Mean survival**	**SE**	*P* ^*^
**RASI**	Q1	42	13 (30.95)	25.900	3.050^**^	0.04
	Q2	46	22 (47.83)	18.380	2.170	
	Q3	44	22 (50)	17.160	2.580	
	Q4	44	28 (63.64)	15.980	2.800	
**SE**	Q1	41	19 (46.34)	20.680	2.660	0.57
	Q2	44	16 (36.36)	22.210	2.570	
	Q3	46	25 (54.35)	17.630	2.500	
	Q4	45	25 (55.56)	17.660	3.340	
**BE**	Q1	44	31 (70.45)	16.350	2.560	0.55
	Q2	44	22 (50)	18.800	2.330	
	Q3	45	17 (37.78)	20.760	2.760	
	Q4	43	15 (34.88)	21.240	2.900	

* *One-way ANOVA*.

** *post-hoc Tukey test showing the significant difference with Q4 group*.

**Figure 4:**
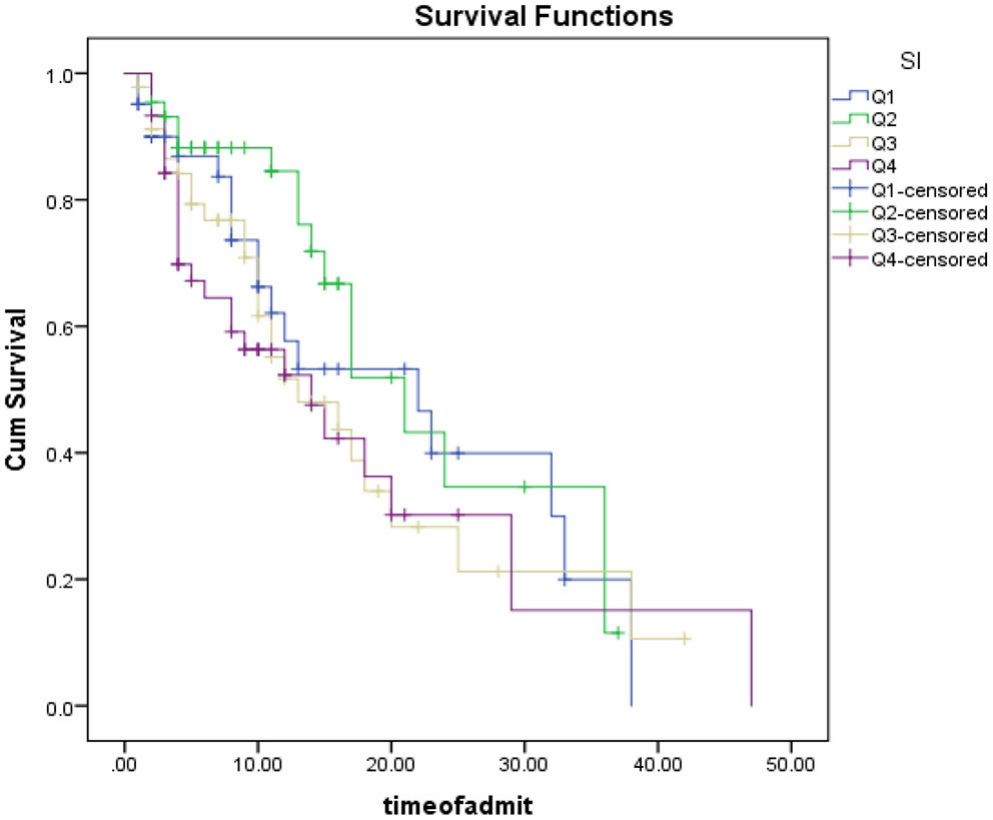
Patient survival analysis Based on SI quarters.

As shown in Figure 4, patients were categorized based on SI quartile into 4 categories <0.76 (Q1), between 0.76 and 0.9 (Q2), between 0.9 and 1.14 (Q3) and more than 1.14 (Q4). Kaplan Mir analysis with log-rank was performed to determine the difference in survival distribution in different SI groups: Survival distribution was not statistically different for the four defined groups, χ2 (3) = 4.31, *p* = 0.320.

**Figure 5:**
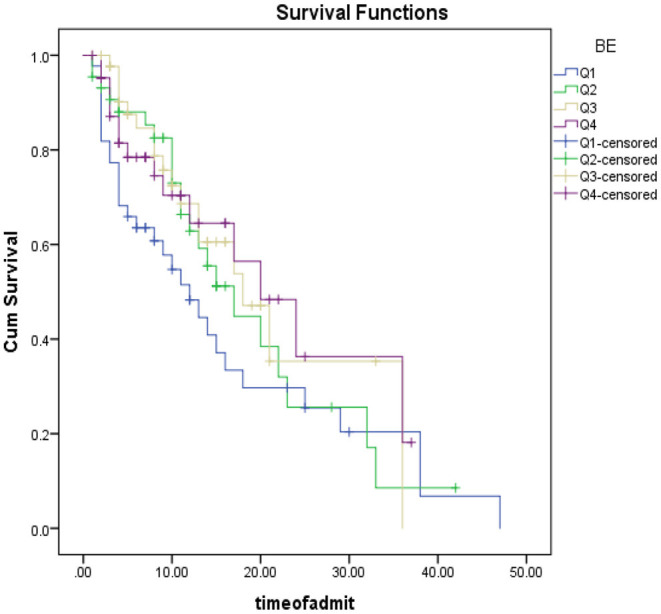
Patient survival analysis based on BE quartiles.

As shown in Figure 5, patients were categorized based on the BE quartile into 4 categories <5- (Q1), between −5 and −0.6 (Q2), between −0.6 and 4.45 (Q3) and more than 4.45 (Q4). Kaplan Mir analysis with log-rank was performed to determine the difference in survival distribution in different SI groups: Survival distribution was not statistically different for the four defined groups, χ2 (3) = 3.87, *p* = 0.275.

**Figure 6:**
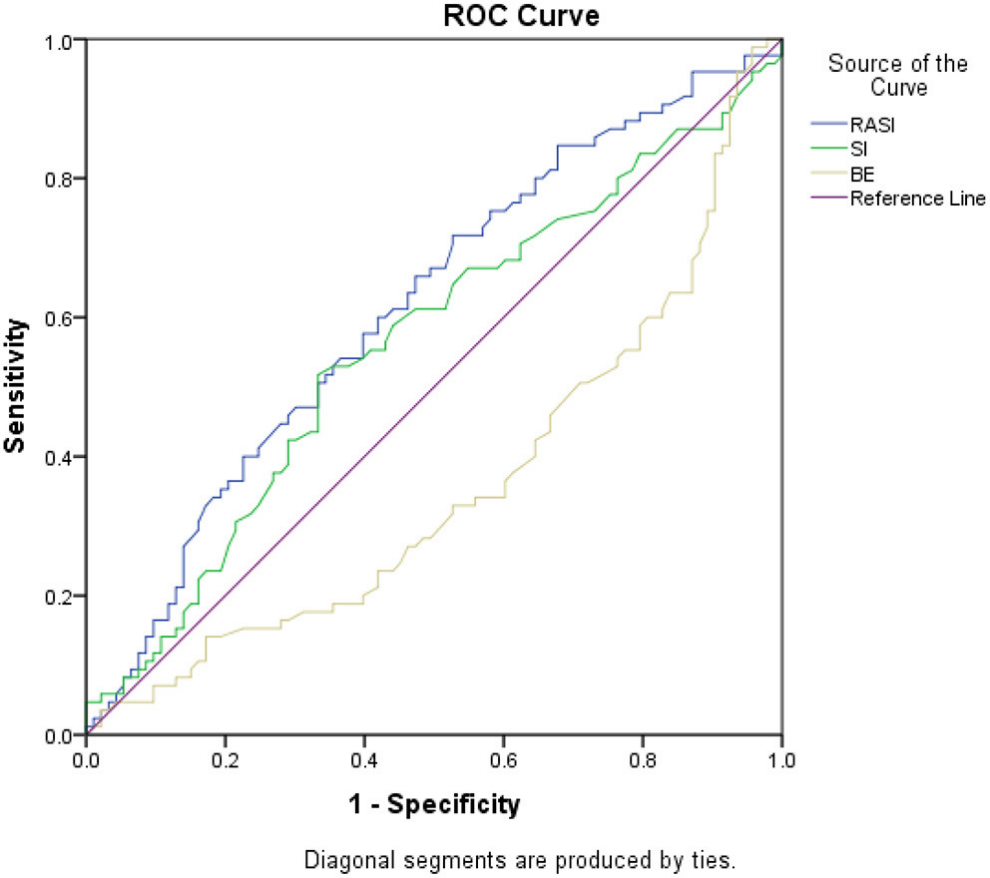
ROC analysis for mortality.

According to Figure 6, in the study of RASI and SI scoring systems, Receiver operator curves analysis showed that the AUC of RASI scoring system for predicting mortality was 0.614, 95% CI (0.531–0.697, *P* = 0.009) and this value was equal to 0.354, 95% CI (0.277–0.441, *P* = 0.001) for BE; while this value was not significant for SI with AUC of 0.562, 95% CI (0.477–0.647, *P* = 0.152); while Delong's test showed no significant difference in AUC of RASI and SI (chi^2^(1) =2.09, *P* = 0.1480). RASI scoring system with 97% sensitivity and 96% specificity predicted mortality with a 1.285 cut-off. The BE scoring system, with a sensitivity of 98.8% and a specificity of 97.8%, predicted mortality with a cut-off of 16.9. Cox analysis showed that none of the variables were associated with mortality (*P*> 0.05); Except for hospitalization in the intensive care unit, which was significantly identified as a risk factor for death with a risk ratio of 6.723 (*p* = 0.002) (Table 4).

**Table 4:** Relationship between demographic variables and mortality.

		**HR**	**95% CI**		***P***
			**Lower**	**Upper**	
Sex, male		0.774	0.476	1.259	0.302
Age		1.012	1.029	0.996	0.137
RASI		0.895	1.985	0.403	0.785
SI		1.841	82.176	0.041	0.753
BE		1.041	1.005	0.969	0.801
RR		1.027	1.11	0.95	0.497
PR		1.003	1.028	0.977	0.847
SBP		0.996	1.017	0.975	0.69
**Level of consciousness**	Normal	0	0	0	Ref
	Decreased	1.47	2.642	0.818	0.197
	Confusion	1.036	2.146	0.5	0.925
**Admission type**	ICU	0	0	0	Ref
	Ward	1.631	3.313	0.803	0.176
	ED	6.723	2.638	17.131	0.002

## Discussion

Sequential Organ Failure Assessment (SOFA) score is being used as a prognostic factor in sepsis, but it requires multiple laboratory indices that would not be rapidly available in the setting of an emergency department (11). This was the reason that many later studies tried to develop easier to use prognostic factors like the quick SOFA as we discussed. This study also aimed at evaluating one of these newly developed scoring systems, RASI.

In the present study, 56.7% of subjects with sepsis were male and 43.3% were female. As reviewed in the study by Angele et al., Numerous experimental and clinical studies have shown gender differences in infectious diseases and sepsis. Females are less likely to develop sepsis and infection, while the male gender may be a risk factor for disease due to decreased cellular immune response and cardiovascular function (12). This was somewhat true in our study as well, and the number of men with sepsis was higher.

86.51% of the patients we studied had pneumo-sepsis. This has been seen in several studies and the most common site of infection leading to sepsis is the lung (64% of cases) (13).

The Systemic Inflammatory Response Scale (SIRS) has conventionally been utilized to monitor for sepsis in emergency department patients (14), but the requirement of using other indexes in this field was felt due to the new diagnostic criteria outlined in the third session of the International Consensus Definitions Task (15). The main purpose of our study was to investigate the relationship between RASI and SI systems and paraclinical data related to BE in predicting the course of sepsis. SI is a criterion that has been used before in predicting the clinical course of treatment of different patients in traumatic injuries (16), gynecological diseases (17), cardiovascular diseases (18), and sepsis.

SI was compared to the SIRS-2 and modified SIRS criteria (SIRS omitting white blood cell count) in a retrospective analysis of 2,524 adult individuals. The 28-day mortality prognosis for SI, SIRS, and modified SIRS was low in their research (19). In our study, the SI criterion was not able to predict mortality in sepsis patients and our study is consistent with this study.

In another study of 295 patients with severe sepsis, SI did not predict the need for vasopressor use or mortality (20). However, this issue was also seen in our study, the need to use vasopressor is a good variable for further studies, which unfortunately was not considered in our study.

But fewer studies have been done on RASI. Our study showed that RASI scores are significantly able to predict mortality in people with sepsis. The study by Jiang et al. showed that the use of RASI in the emergency department was able to predict the incidence of sepsis in patients with suspected sepsis (10). In their study, respiration rate was integrated into SI to increase the RASI predictive ability to identify patients with sepsis, which was ultimately more sensitive to lactate alone as well as to other screening tools. They showed that RASI was significantly able to predict discharge or hospitalization status. However, in their study, the final outcome of treatment was not followed up. However, our study examined mortality in these patients. It can be said that one of the advantages of our study in choosing the final outcome was the absence of confounding factors related to the physician's decision to admit or discharge the patient.

However, it seems that no other study has been done on the use of this index in sepsis patients in order to compare the results of the present study with it. But it is noteworthy that in our study RSAI had a significant inverse correlation with RR (Spearman rho = −0.486; *P* < 0.001). The median number of respiration per minute in our patients was 30.32 (24-09.5) which could also be interpreted according to the RASI calculation formula [HR / SBP ^*^ (RR / 10)].

In our study, no significant relationship was found between BE and clinical outcome, but other studies found acidosis assessed by BE and/or pH to be promising for predicting risk in septic patients. In the study by Wernly et al. (21), in contrast to our study, BE was an independent predictor of mortality. However, they used BE values along with the pH index in their analyzes, which may be the reason why their study differs from ours. But in another study by Gattinoni et al., “Alactic BE,” meaning the total concentration of lactate and negative BE, was not useful in predicting mortality in patients with sepsis (22). Which is somewhat consistent with our study. But according to the research, lactate metabolism is complex, and lactate levels may be close to “normal” even in patients at risk of death and adverse outcomes, and much more research is needed to make that decision.

## Study Limitations

One limitation was that the sample size of the subjects in our study was low. However, other limitations in not considering other useful parameters such as lactate and pH made it difficult to interpret our results for the BE index; So considering the BE and pH, assessing the base acid balance and buffer capacity may help us and increase our ability to predict mortality risk. Unfortunately, we did not collect all data needed for the calculation of SOFA score. A comparison of SOFA and RASI could be assessed in further studies. One main limitation of this study was the matter of linearity in statistical analyses. While logistic regression does not require a linear relationship between the dependent and independent variables; we assumed linearity for adjusting potential confounding factors in the regression model. This Intention-to-treat approach might give different results getting adjusted for different variables. But an alternative approach to machine learning, entitled ensemble modeling, is proposed by Zhang et al. that could be used in further studies (23).

## Conclusion

The results of our study showed that RASI scoring alone could be a good criterion for predicting the chance of mortality in patients with sepsis, while is not superior to previous criteria of SI and could be used complementary. Patients with high RASI scores should be given more attention to reducing the chance of death.

## Suggestions

This study was performed in only one center with a small number of patients. It is suggested that further studies with larger sample sizes be performed to confirm the findings of the present study. Also, in advance, other factors such as lactate levels and pH are also assessed in the study. Checking RASI during hospitalization and its relationship with other indicators can also be helpful.

## Data Availability Statement

The original contributions presented in the study are included in the article/supplementary material, further inquiries can be directed to the corresponding author.

## Ethics Statement

The studies involving human participants were reviewed and approved by Mashhad University of Medical Sciences, Mashhad, Iran. Written informed consent for participation was not required for this study in accordance with the national legislation and the institutional requirements.

## Author Contributions

FM and MF designed the study. Data was collected by FB, SA, and MF. MF, FB, SA, and AMH collected the data. MF and AMH analyzed the data. The draft of the manuscript was provided by FM, FB, SA, AMH, and MF. AMH, ZB, RF, and HZM edited the manuscript in revisions. All authors confirmed the final format. All authors listed have made a substantial, direct, and intellectual contribution to the work and approved it for publication.

## Conflict of Interest

The authors declare that the research was conducted in the absence of any commercial or financial relationships that could be construed as a potential conflict of interest.

## Publisher's Note

All claims expressed in this article are solely those of the authors and do not necessarily represent those of their affiliated organizations, or those of the publisher, the editors and the reviewers. Any product that may be evaluated in this article, or claim that may be made by its manufacturer, is not guaranteed or endorsed by the publisher.
